# Construction and analysis of a competing endogenous RNA network to reveal potential prognostic biomarkers for Oral Floor Squamous Cell Carcinoma

**DOI:** 10.1371/journal.pone.0238420

**Published:** 2020-09-15

**Authors:** Wenjing Zhang, Shuai Xu, Laner Shi, Zhangzhi Zhu, Xinying Xie

**Affiliations:** 1 Department of Health Management Center, The Third Affiliated Hospital, Southern Medical University, Guangzhou, Guangdong, China; 2 Department of First College of Clinical Medicine, Guangzhou University of Traditional Chinese Medicine, Guangzhou, Guangdong, China; 3 Endocrine Department, Shenzhen Bao'an Traditional Chinese Medicine Hospital, Guangzhou University of Chinese Medicine, Shenzhen, Guangdong, China; 4 Endocrine Department, Guangzhou University of Traditional Chinese Medicine First Affiliated Hospital, Guangzhou, Guangdong, China; 5 General Department, Guangzhou University of Traditional Chinese Medicine First Affiliated Hospital, Guangzhou, Guangdong, China; Tekirdag Namik Kemal University, TURKEY

## Abstract

**Background:**

Patients diagnosed with Oral Floor Squamous Cell Carcinoma (OFSCC) face considerable challenges in physiology and psychology. This study explored prognostic signatures to predict prognosis in OFSCC through a detailed transcriptomic analysis.

**Method:**

We built an interactive competing endogenous RNA (ceRNA) network that included lncRNAs, miRNAs and mRNAs. Gene Ontology (GO) and Kyoto Encyclopedia of Genes and Genomes (KEGG) were used to predict the gene functions and regulatory pathways of mRNAs. Least absolute shrinkage and selection operator algorithm (LASSO) analysis and Cox regression analysis were used to screen prognosis factors. The Kaplan-Meier method was used to analyze the survival rate of prognosis factors. Risk score was used to assess the reliability of the prediction model.

**Results:**

A specific ceRNA network consisting of 56 mRNAs, 16 miRNAs and 31 lncRNAs was established. Three key genes (HOXC13, TGFBR3, KLHL40) and 4 clinical factors (age, gender, TNM, and clinical stage) were identified and effectively predicted the for survival time. The expression of a gene signature was validated in two external validation cohorts. The signature (areas under the curve of 3 and 5 years were 0.977 and 0.982, respectively) showed high prognostic accuracy in the complete TCGA cohort.

**Conclusions:**

Our study successfully developed an extensive ceRNA network for OFSCC and further identified a 3-mRNA and 4-clinical-factor signature, which may serve as a biomarker.

## Introduction

Oral cancer is one of the most common malignant tumors of the head and neck [1]. Only 10% of patients with advanced metastatic oral cancer survive for 5 years following therapy [2]. The incidence of Oral Floor Squamous Cell Carcinoma (OFSCC) is only inferior to tongue cancer. Surgery is the main radical treatment [3].

MiRNA combined with targeted mRNA and render it untranslatable normally, silencing the corresponding genes. In 2011, Salmena [4] proposed the competitive endogenous RNA (ceRNA) hypothesis, which states that lncRNA could combine competitively with miRNAs, thus eliminating the inhibition of miRNAs on target genes and regulating the expression of target genes.

Therefore, the pathogenesis of ceRNA has attracted considerable attention. In many malignant tumors, such as colon cancer, liver cancer and lung adenocarcinoma, the mechanism of tumorigenesis and development induced by lncRNA-miRNA-mRNA has been elucidated [5].

In the present study, mRNA and mature miRNA material of patients with oral floor OSSC were collected and analyzed. A ceRNA network was designed. Meanwhile, a functional analysis of mRNAs from the ceRNA network was performed. We identified 3-mRNA as novel candidate biomarkers for OFOSSC. The mRNA expression profiles were combined with the clinical features, as the potential model to predict survival.

## Materials and methods

### Patients and TCGA data retrieval

The publicly available TCGA dataset (https://portal.gdc.cancer.gov/) concentrates cancer genetic information from previous studies. This initiative is of considerable significance in the diagnosis and prevention of cancer in future generations. Because all data in this paper were extracted from TCGA, this study strictly followed the publication guidelines approved by TCGA (https://cancergenome.nih.gov/publications/publicationguidelines), and an ethics application was not required. The RNA sequence data (mRNA and lncRNA; Illumina HiSeq RNA-Seq platform), mature miRNA sequence information (Illumina HiSeq miRNA-Seq platform), and clinical data of 54 samples from OFSCC and 3 samples from normal tissues were retrieved and downloaded from TCGA (up to Aug 29, 2019). (Refer to S1 File for retrieval and download files). The RNA material was merged by Perl (See “6.merge_files.pl” in the S1 File) and transformed into lncRNAs (sense_overlapping, lncRNA, 3prime_overlapping_ncrna, processed_transcript, antisense, sense_intronic) and mRNAs (protein coding) through the Ensembl database (http://www.ensembl.org/index.html, version 89) [6]. Ensembl is a software system capable of automatic annotation and maintenance of eukaryotic genomes. Mature miRNA data manual was extracted and merged by Perl (See “6.merge_files.pl” in the S1 File).

(See the " 7. The RNAs (lncRNAs, miRNAs, mRNAs) expression of the studied samples" folder of S1 File for the sorted data.)

### Differential analysis of DERNAs

Differentially expressed RNAs (DERNAs) contain mRNA, miRNA and lncRNA. The Ensembl database was used to separate lncRNAs from mRNAs. We normalized the classified data and conducted gene identification. The false discovery rate (FDR) was used to correct the statistical significance of the multiples test. Log2 fold changes(log2FC) > 1 and FDR < 0.05 were considered to be significant. All of the above operations were implemented by the edgeR software package [7]. Then, we generated volcano plots with three types of DERNAs obtained from the previous step using the pilots package.

### Construction of a ceRNA network

MiRcode was used to predict the targeted DEmiRNAs of DElncRNAs [8]. Three databases, Targetscan (http://www.targetscan.org/), miRDB [9] (http://www.mirdb.org/) and miRTarBase [10], were used to predict the target mRNAs of DEmiRNAs by combined utilization. The intersection of DEmRNAs and targeted mRNAs was perceived by the VennDiagram software package. The visualization of coexpression constructed by cytoscape 3.7.1. revealed the potential relationship of RNAs in OFSCC

### Functional enrichment analysis

To further analyze the functional characteristics of DEmRNAs in OFSCC, GO enrichment and KEGG pathway analyses were performed based on the Database for Annotation, Visualization and Integrated Discovery (DAVID) bioinformatics resources (version 6.8) https://david.ncifcrf.gov/ [11]. A significance level of P< 0.05 was set as the cutoff criteria.

### Survival analysis and a predictive model for prognosis construction

We used the “survival” package in R software for Kaplan-Meier (K-M) survival analysis of DEmRNAs in the ceRNA network.

The DEmRNAs were evaluated using univariate Cox’sproportional hazard regression model. Genes with P-value <0.05 were considered as candidate variables and entered into a stepwise LASSO regression [12–15] and multivariate Cox regression analysis.

### Risk assessment models and ROC curve construction

The risk score was the linear combination of key genes weighted by the regression coefficients. A risk score of patients was determined on the basis of the following Equation.

Riskscore=Exp1*Coe1+Exp2*Coe2+Exp3+Coe3+……Expi*Coei.

In this Equation, Exp represents the value of expression of key genes, and Coe represents their corresponding coefficients from the multivariable Cox regression analysis. Finally, we established a prognostic risk score system based on screen-out of mRNAs. A time-dependent receiver operating characteristic (ROC) curve was used to estimate the specificity and sensitivity of the prognosis factors in predicting survival and to estimate the distinguishing and predictive abilities of the risk score system. The AUC of ROC was calculated to predict the 3- or 5-year survival rate of patients with OFSCC.

### Validation of the key genes in GEO dataset

Three GEO Datasets (GSE74530 with 6 normal samples and 6 tumor samples, GSE30784 with 45 normal samples and 167 tumor samples, GSE23558 with 5 normal samples and 27 tumor samples) provided external validation of key genes. The *adj*.*P* value, log2FC and expression of the key genes in both the TCGA and three GEO datasets were calculated by the limma package.

### Combining the key genes with clinical characteristic prediction for OFSCC

To evaluate the prognostic value of different clinical characteristics, such as age, gender, clinical_stage, TNM, invasive degree (T stage), lymph node status (N stage), metastasis (M stage), and risk scores of key genes, a Cox proportional hazards model was constructed to investigate the prediction capability of prognosis in OFSCC. The result of the multivariable Cox analysis was displayed by nomogram, which was created with the R package rms. Then, risk score and the ROC curve were used to evaluate the accuracy of the prediction model. Moreover, we investigated the potential prediction ability of prognosis in OFSCC by combining risk score and clinical characteristics (age + stage) using a Kaplan-Meier estimator and log-rank test.

## Results

### Differentially expressed mRNAs in OFSCC

**Fig 1:**
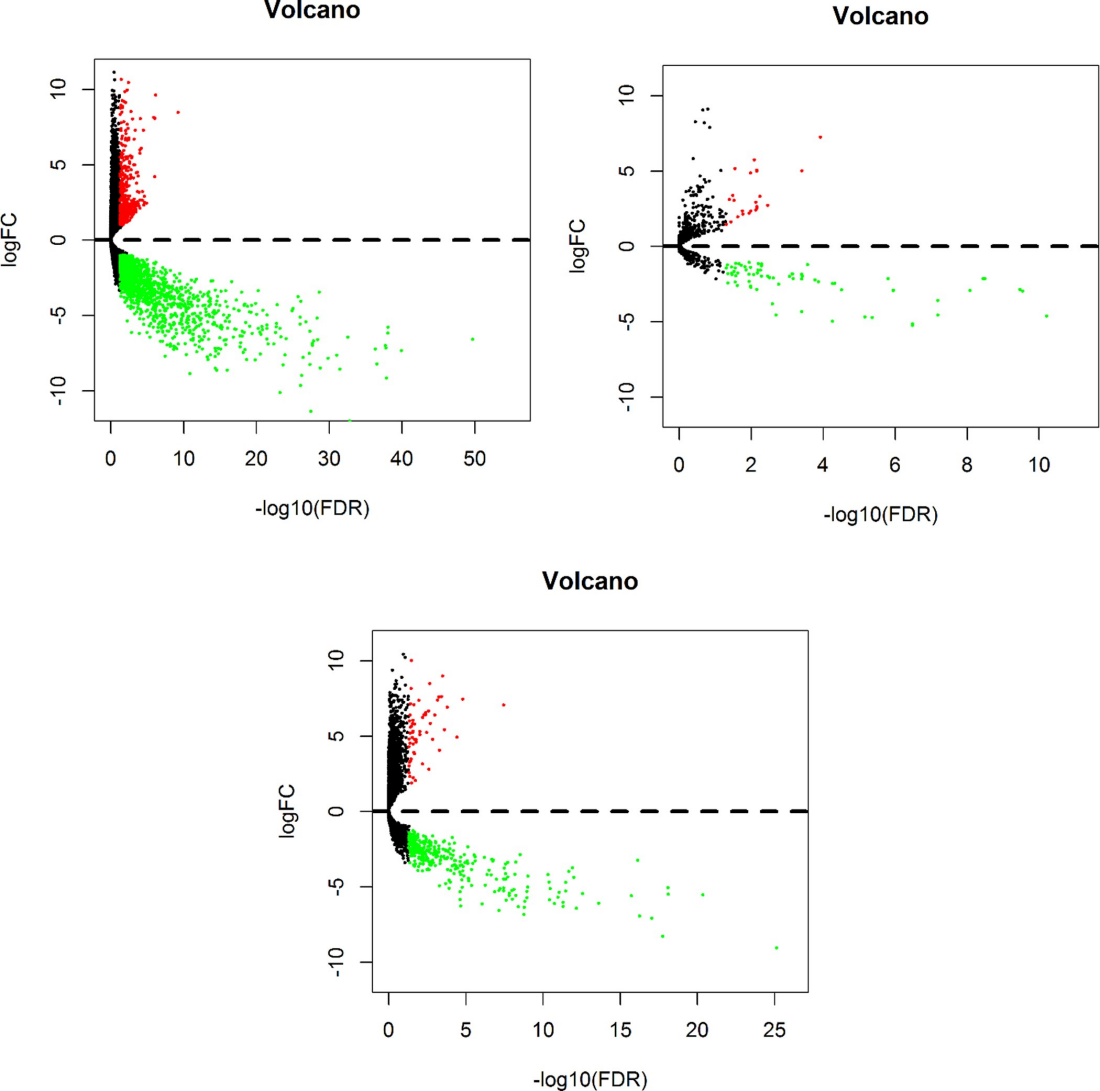
Volcano maps of differentially expressed RNAs in OFSCC patients. (A) mRNA (B) lncRNA (C) miRNA. The red dot represents up‐regulated RNA, and green dot represents down‐regulated RNA. log2|FC| > 1.0 and P < 0.05 as the selection criteria. OFSCC: Oral Floor Squamous Cell Carcinoma; FC: fold change; lncRNAs: long noncoding RNAs; miRNA: microRNA; mRNA: messenger RNA.

The significant genes of DERNAs were screened out using log2FC> 1 and P< 0.05 by volcano map. A total of 2198 DEmRNAs (543 upregulated and 1655 downregulated), 92 DEmiRNAs (23 upregulated and 69 downregulated), and 372 DElncRNAs (58 upregulated and 314 downregulated) between 54 tumor tissues and 3 normal tissues were identified (Fig 1).

### Construction of the ceRNA network

**Fig 2:**
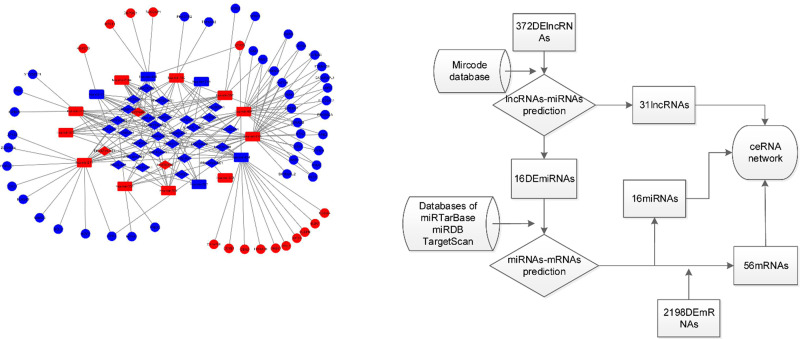
(A) The ceRNA regulatory network in OFSCC. The nodes highlighted in red indicate expression up-regulation, and the nodes highlighted in blue indicate expression down-regulation. LncRNAs, miRNAs and mRNAs are represented by diamonds, rounded rectangles, and squares, respectively. (B) A Flow chart for ceRNA network. ceRNA: competitive endogenous RNA.

To further understand the role of DERNAs in OFSCC and to further elucidate the interactions between these DEmRNAs, DElncRNAs and DEmiRNAs, we constructed a ceRNA regulatory network of DERNAs step by step. Step 1: We predicted the miRNAs targeted by the 372 DElncRNAs using the miRcode database. Step 2: We predicted that 16 miRNAs could interact with the 31 lncRNAs. Step 3: A total of 595 miRNA-targeted mRNAs were extracted from three databases (miRTarBase, miRDB and TargetScan). Step 4: A total of 71 DEmRNAs were obtained from the intersection of 595 targeted mRNAs and 2198 DEmRNAs. Step 5: A total of 56 mRNAs were obtained with the reverse trend of related miRNA expression. We constructed the ceRNA network of OFSCC using 56 mRNAs, 16 miRNAs and 31 lncRNAs. Flow chart and network diagrams are shown in Fig 2.

### GO and KEGG analyses

**Fig 3:**
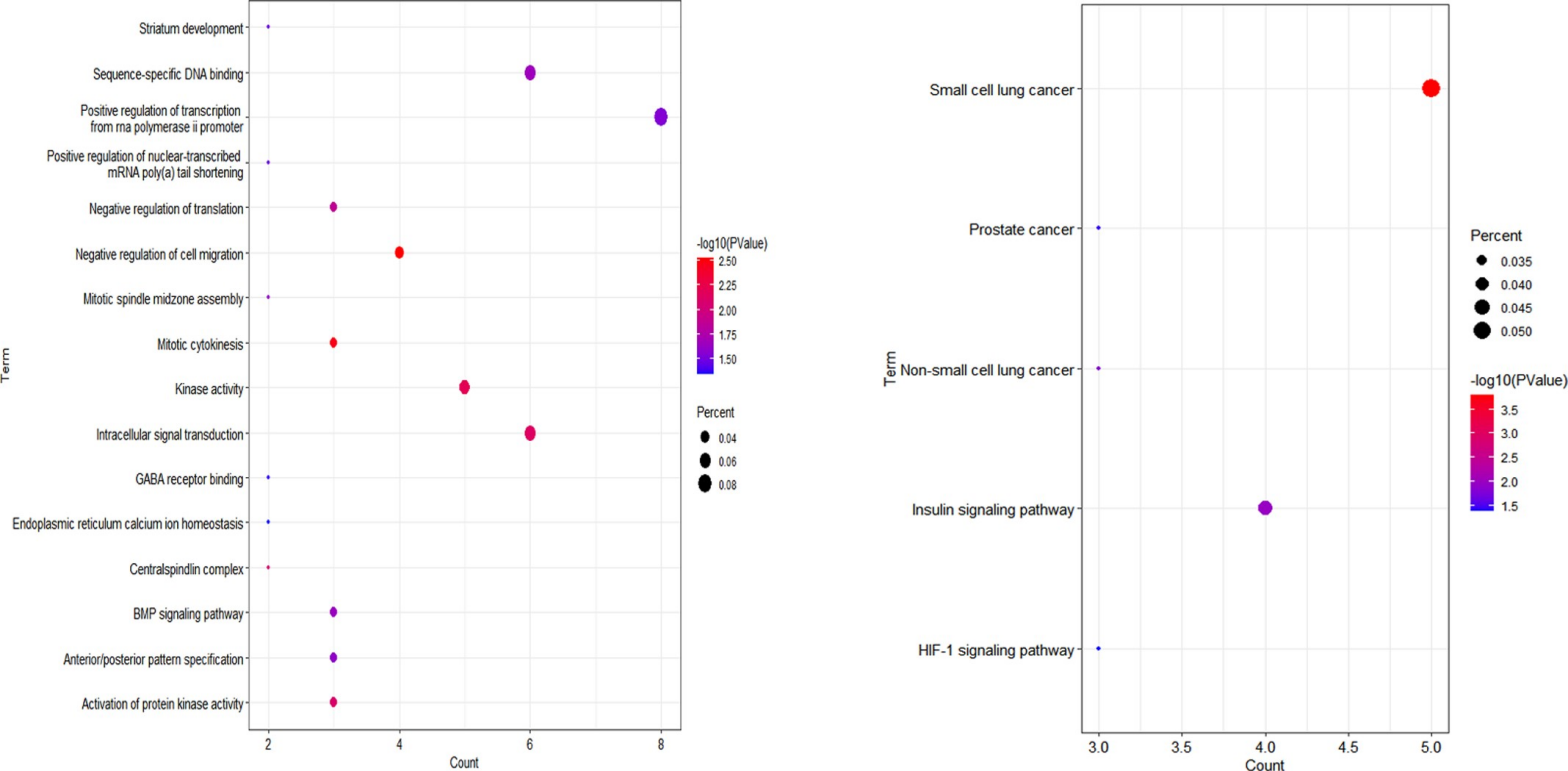
Enrichment analysis of GO (A) and KEGG (B). The x‐axis indicates the number of DEmRNAs participating in the given GO term and pathway. GO: Gene Ontology.

In addition, to elucidate the mechanisms underlying OFSCC and to further understand the functional characteristics of DEmRNAs, GO and KEGG analyses were performed via the DAVID and ggplot2 packages of R software. 16 significantly enriched GO terms and 12 significantly enriched KEGG pathways are listed in Fig 3 and S1 and S2 Tables.

### Key genes were screened out by comprehensive analysis

**Fig 4:**
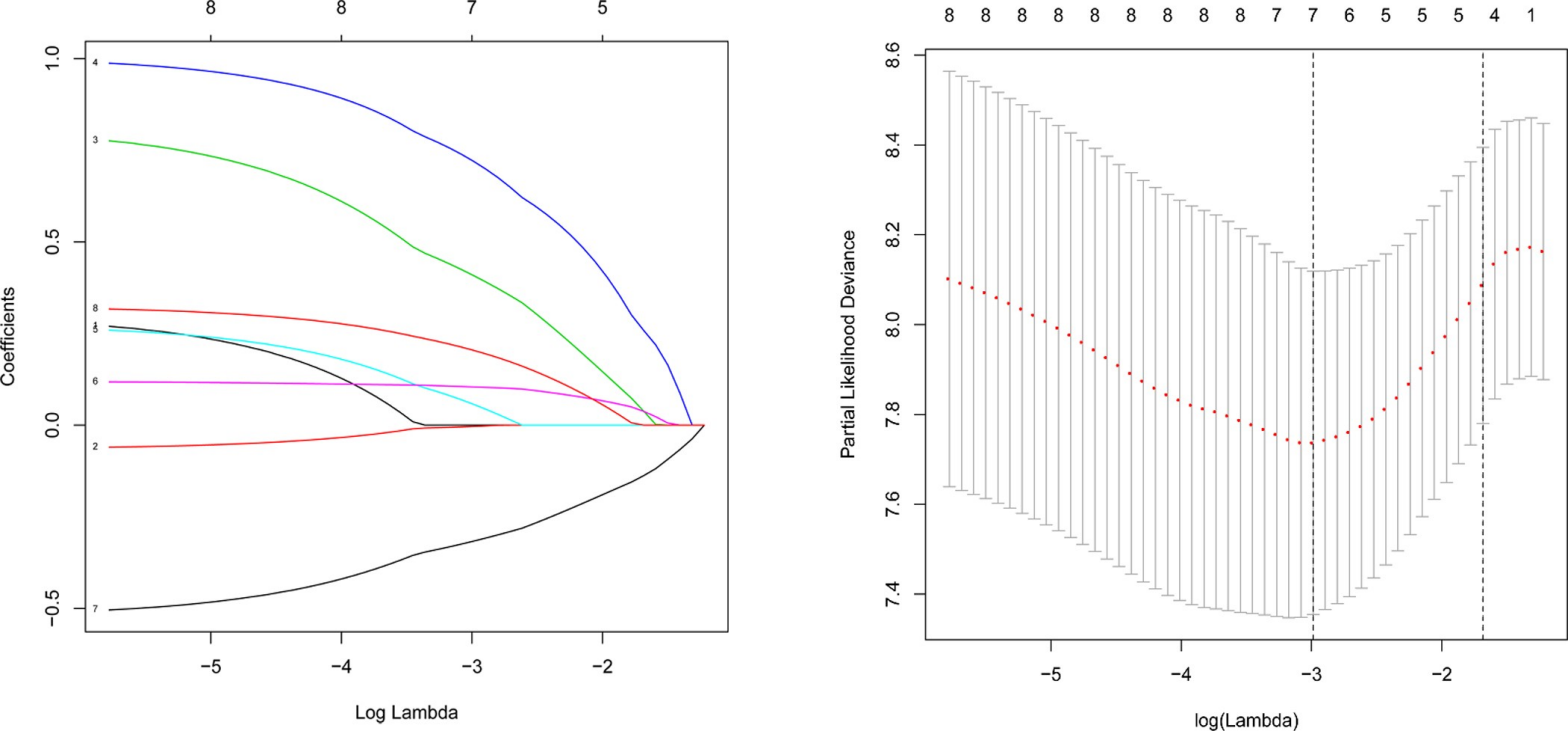
Lasso‐penalized Cox regression analysis of 9 DEmRNAs. The coefficient values at varying levels of penalty (A). Each curve represents an mRNA. Ten‐fold cross‐validation was used to calculate best lambda which leads to minimum mean cross‐validated error (B). Red dots represent partial likelihood deviance; solid vertical lines indicate their corresponding 95% CI; the left dotted vertical line is the value of lambda that gives minimum cvm, named lambda. min; the right dotted vertical line is the largest value such that error is within 1 standard error of the minimum, named lambda. 1se.

**Fig 5:**
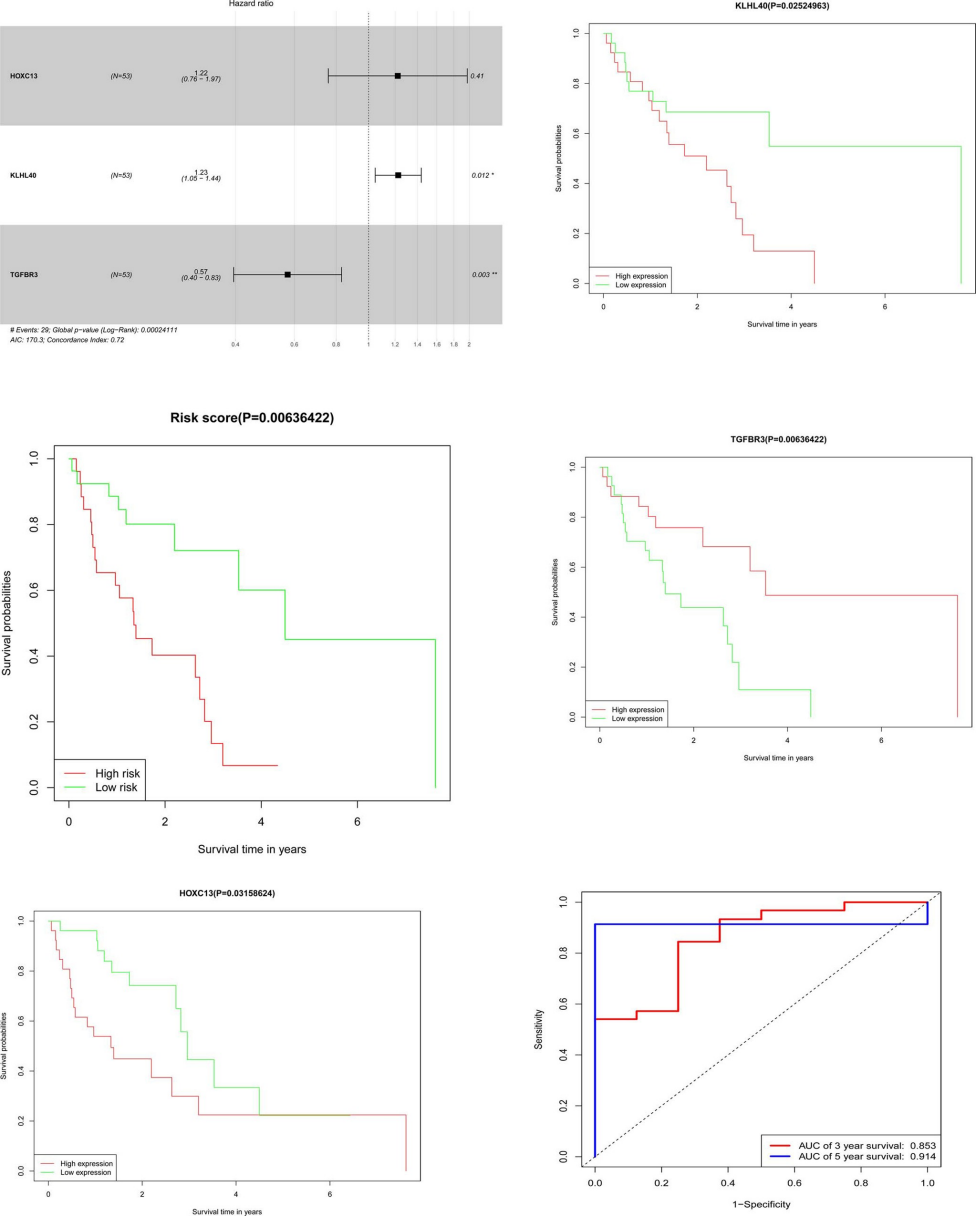
(A) Multivariate Cox regression analysis in patients with OFSCC, (B) Risk score analysis of 3 key mRNA signatures, (C, D, E) Kaplan-Meier survival curves for key mRNAs, (F) ROC curve of risk score for predicting 3-y and 5-y survival.

Only 9 of 56 mRNAs in ceRNA network were statistically significant though unvariable Cox analysis. Further, 7 mRNAs with statistical difference were screened out, though lasso analysis (Fig 4). So we carried out survival analysis on 7 mRNA respectively, and found that 4 mRNAs had statistical differences, which were TGFBR3, KLHL4, HOXC13 and HADHB. Though literature analysis, we selected these three mRNAs as key genes for verification (S1 File). TGFBR3, KLHL40, and HOXC13 were screened out, and a predictive mRNA model was constructed (Fig 5A and Table 1). The risk score based on mRNA expression according to their relative coefficient in the multivariate Cox regression was calculated as: Risk score = ExpHOXC13*0.16163+ExpKLHL40*0.19592+ExpTGFBR3*(-0.52294).

**Table 1:** Result of multivariate Cox regression.

mRNA	HR (95%CI)	p Value
**HOXC13**	1.2229(0.7578–1.9733)	0.40984
**KLHL40**	1.2268(1.0468–1.4377)	0.01155*
**TGFBR3**	0.5728(0.3951–0.8303)	0.00327**

We calculated the risk score for each patient. With the median as the critical value, patients were divided into high-risk and low-risk groups. (S3 Table and Fig 5B) Survival analysis of 3 key genes was performed using the Kaplan-Meier method with a log-rank statistical test. (Fig 5C–5E) The ROC curve shows that 3 key genes exhibited relatively accurate prognostic capacity for predicting survival rates in OFSCC with AUCs of 0.853 (3-year) and 0.914 (5-year). (Fig 5F)

### Validation of the expression of key gene molecules with GEO data

**Fig 6:**
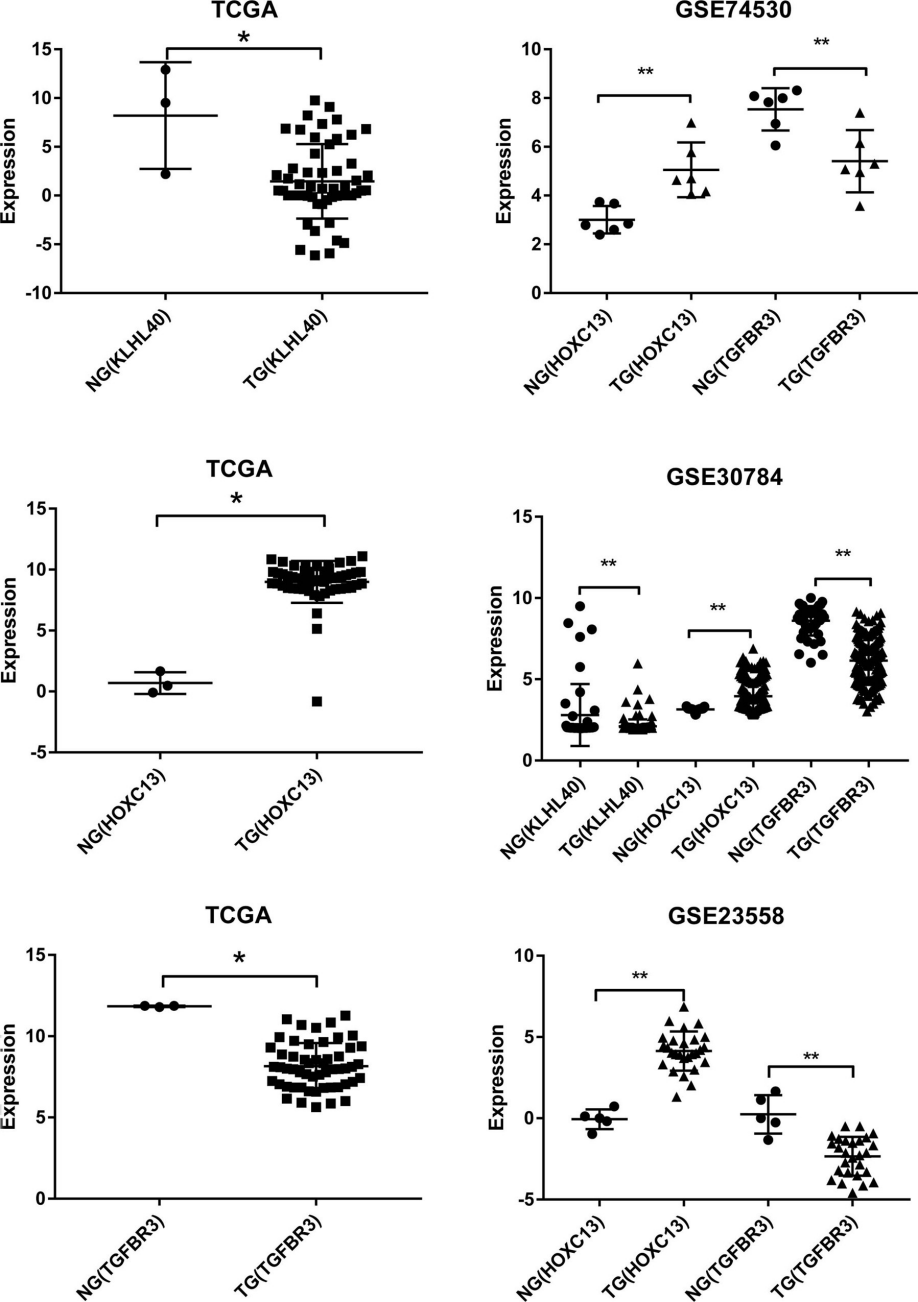
(A, B, C) The expression of 3 key mRNAs in TCGA. (D, E, F) The expression verification of 3 GEO databases.

Three studies were screened out from GEO to verify the differential expression of key mRNAs in OFSCC. As shown in Fig 6 and Table 2, the expression trend of the 3 molecules essentially is consistent with our results.

**Table 2:** The expression trend of the 3 molecules.

	Normal	Tumor	mRNA	logFC	*P*.Value	*adj*.*P*.Val
**TCGA**	3	54	KLHL40	-5.78652951	3.914E-08	1.4141E-06
			HOXC13	8.464825534	9.5568E-12	5.8414E-10
			TGFBR3	-2.96896265	7.9632E-08	2.7759E-06
**GSE74530**	6	6	KLHL40	-0.02306571	1	1
			HOXC13	2.037654	0.000479	0.010496
			TGFBR3	-2.34335	0.000476	0.010471
**GSE30784**	45	167	KLHL40	-0.69654	0.000024	0.0000891
			HOXC13	0.81523	4.71E-08	2.61E-07
			TGFBR3	-2.47574	4.51E-23	1.54E-21
**GSE23558**	5	27	KLHL40	-0.369703	0.492266	0.702145
			HOXC13	3.840817	3.35E-08	0.0000111
			TGFBR3	-2.15835	0.001284	0.016527

### Combining risk score with clinical significance prognostic prediction for OFSCC

Based on the clinical data, 4 clinical (age, gender, TNM, clinical stage) factors were selected, which may affect the prognosis of the cancer. We further combined the clinical features with the risk level of three mRNAs to construct a prognostic model and evaluated the influence of the above factors on the survival time by multivariate Cox regression analysis. In the multivariate analysis, age (age 50–59, age 60–69, age 70–79, age > = 80), gender, T stage, N stage, M stage, clinical stage, and 3 mRNA signatures were associated with the survival time (Table 3). Additionally, we performed a log-rank test and Kaplan-Meier survival analysis for 3 key genes and clinical features to obtain factors that exhibit a close relation to the prognosis and survival time of patients with OFSCC.

**Table 3:** The multivariate analysis of both clinical factors and 3 mRNA signatures.

	HR	lower .95	upper .95	z	Pr(>|z|)
**risk_level**	5.12E-02	1.75E-02	1.50E-01	-5.419	5.99e-08 ***
**age50-59**	3.23E+00	1.12E+00	9.32E+00	2.17	0.03000 *
**age60-69**	3.65E+00	9.82E-01	1.35E+01	1.933	0.05325.
**age70-79**	1.15E+00	4.01E-01	3.29E+00	0.257	0.79727
**age> = 80**	1.10E+02	2.32E+01	5.19E+02	5.93	3.03e-09 ***
**sexMale**	5.94E+00	1.79E+00	1.97E+01	2.915	0.00356 **
**MM1**	7.26E+05	1.53E+05	3.44E+06	17.009	< 2e-16 ***
**NN1**	3.43E+02	1.32E+02	8.90E+02	12.004	< 2e-16 ***
**NN2**	3.20E-07	3.53E-08	2.90E-06	-13.294	< 2e-16 ***
**NN2a**	2.21E+00	2.28E-01	2.14E+01	0.685	0.49333
**NN2b**	3.09E+01	6.76E+00	1.41E+02	4.426	9.60e-06 ***
**NN2c**	6.55E+00	1.79E+00	2.39E+01	2.839	0.00452 **
**TT2**	7.70E+07	1.60E+07	3.71E+08	22.654	< 2e-16 ***
**TT3**	6.22E+07	2.25E+07	1.72E+08	34.645	< 2e-16 ***
**TT4**	3.52E+05	4.40E+04	2.82E+06	12.035	< 2e-16 ***
**TT4a**	1.21E+06	4.90E+05	2.98E+06	30.394	< 2e-16 ***
**clinical_stage Stage III**	3.75E+04	1.21E+04	1.17E+05	18.214	< 2e-16 ***
**clinical_stage Stage IVA**	1.16E+08	4.43E+07	3.02E+08	37.905	< 2e-16 ***
**clinical_stage Stage IVC**	1.58E+17	1.74E+16	1.43E+18	35.202	< 2e-16 ***

**Fig 7:**
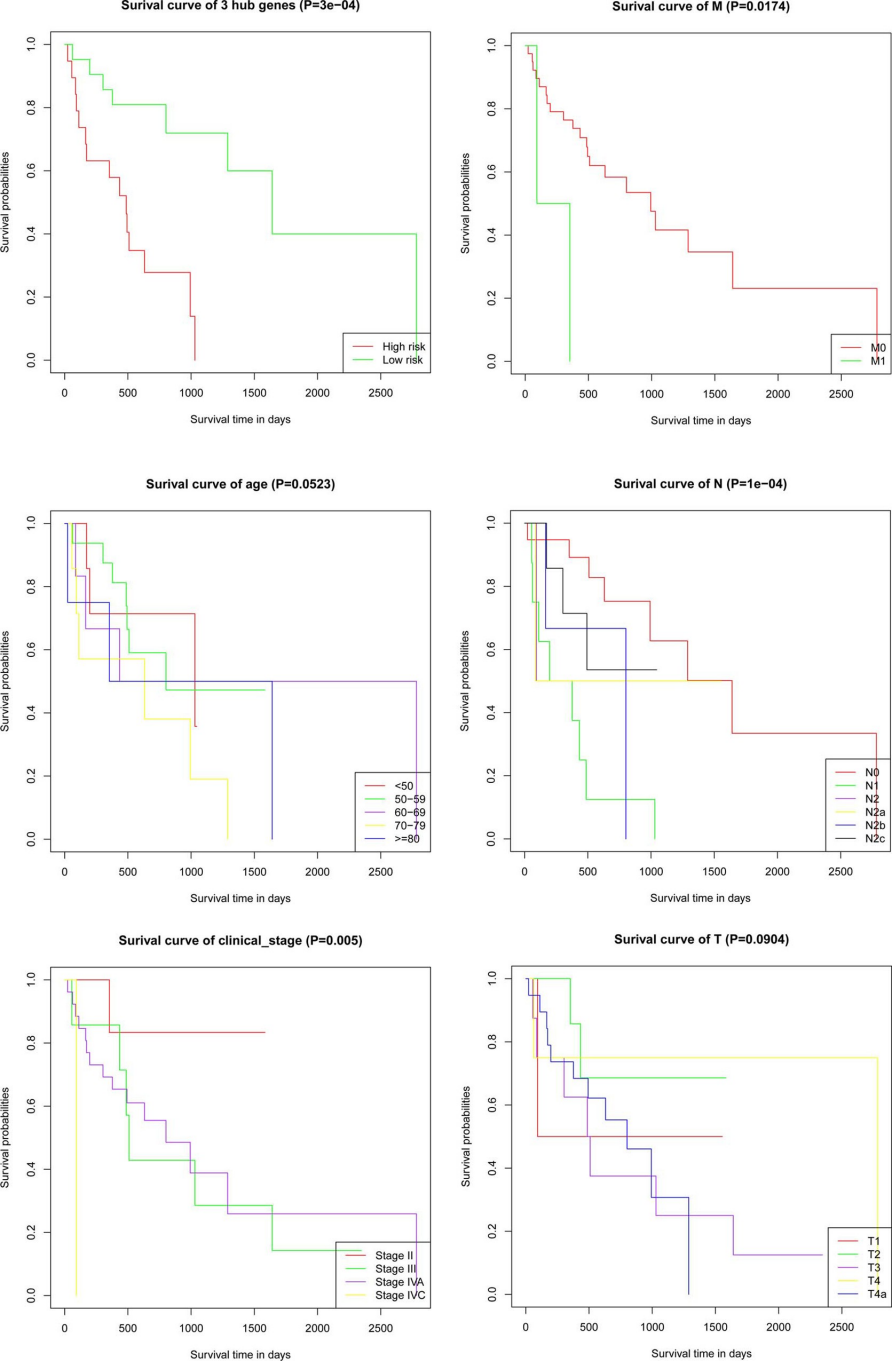
Screening of 3 key genes (A) and prognosis-related clinical characteristics by Kaplan-Meier analyses. (B) Kaplan-Meier curves based on different age groups. (C) Kaplan-Meier curves based on different clinical_stage. (D) Kaplan-Meier curves based on different M stages. (E) Kaplan-Meier curves based on different N stages. (F) Kaplan-Meier curves based on different T stages.

**Fig 8:**
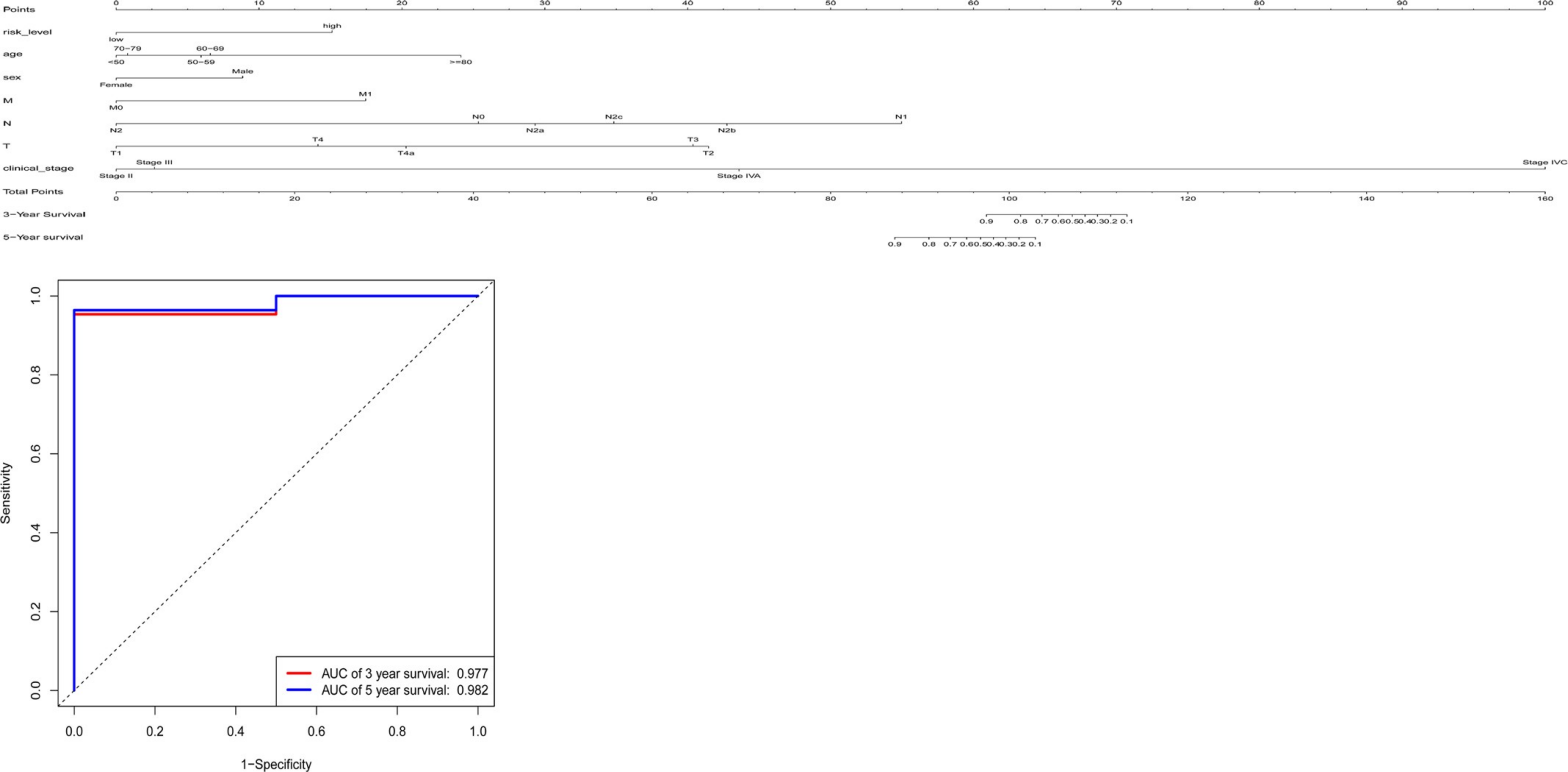
(A) Nomograms to predict 3-y and 5-y survival probability. Total points were calculated by adding up the corresponding points of each individual covariate on the points scale. Then 3-y and 5-y related survival probabilities were obtained by directly converting total points.(B) ROC of both clinical features and 3 key mRNAs.

Kaplan-Meier curves also showed that patient prognosis separated by the risk levels of 3 key genes, N stage, M stage and clinical stage was significantly different (Fig 7). Patients with lower risk score and tumor grade have obviously better prognosis. We constructed a nomogram that integrated the risk score of a model with 3 mRNAs and clinicopathological features to predict the survival probability of patients with OFSCC. We calculated concordance indexes (c-indexes). The C-index quantified the discrimination between random patients, with a C-index of 0.5 indicating no discrimination and 1 indicating perfect discrimination. The C-index for the model was 0.901 (95% CI: 0.8422–0.9598), and the area under the ROC for 3 years was 0.977, and 5 years was 0.982. Both the C-index and the ROC analysis suggested a good predictive performance. (S3 Table and Fig 8)

**Fig 9:**
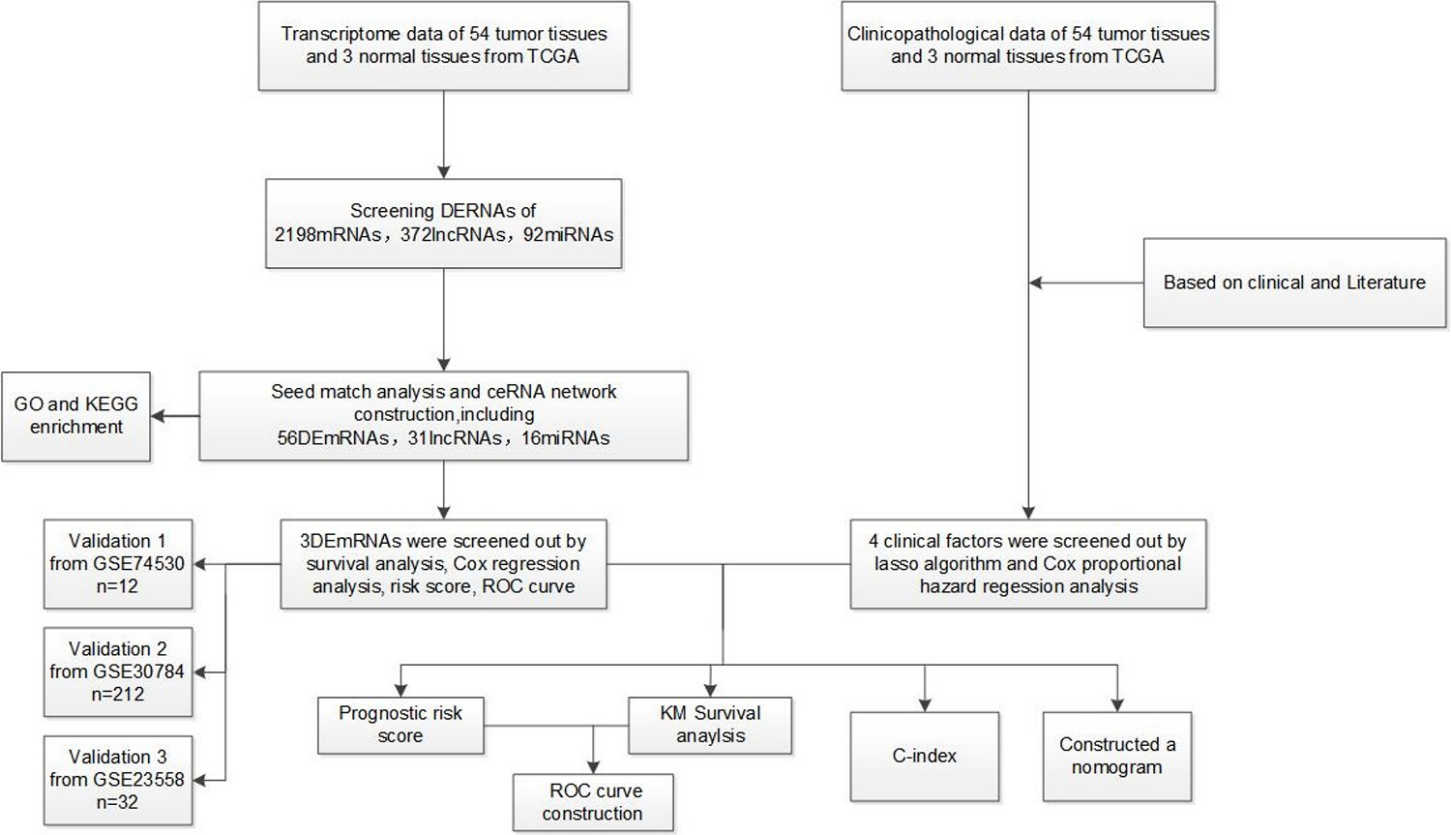
Flow chart of the bioinformatics method.

A flow chart of this method can been seen in Fig 9.

## Discussion

The initial stage of OFSCC is usually asymptomatic, therefore early diagnosis is our priority to optimizing survival rate and the life quality of patients. OFSCC is a molecularly heterogeneous disease, and there are few single genetic driver factors that retain the dominant position in malignant invasive disease identification [16, 17]. Therefore, it is essential for earlier detection and targeted treatment options optimization to novel molecular network biomarkers construction.

It generally accepted that ceRNA alters gene expression through a mechanism mediated by miRNAs, thereby affecting cell function, which may lead to cancer [18]. We established a ceRNA network including 74 mRNAs, 31 lncRNAs and 16 miRNAs with a series of bioinformation datasets. 3-mRNA (TGFBR3、KLHL40 and HOXC13) model was identified in the network and that was associated with the clinical outcome of floor of mouth cancer according to univariate and multivariate Cox proportional regression analyses. In this study, we developed a predictive model based on three mRNAs and four clinical outcomes, and the reliability of the model was also proven. In prostate cancer, breast cancer, renal cell carcinoma and endometrial cancer, TGFBR3 has been identified as a tumor suppressor gene [19–24]. Wei Z found that hsa_circ_0042666 regulated laryngeal squamous cell carcinoma (LSCC) cell proliferation and invasion by the miR-223/TGFBR3 axis [25]. HOXC13 has been reported to be correlated with progression from leukoplakia to OFSCC arising in the Gingivo Buccal Complex (GBC) with integrative genome-wide analysis [26]. HOXC13 was identified as the novel oral cancer driving genes. KLHL40-deficient patients were observed to phenocopy muscle abnormalities. The relationship between it and cancer has not been previously confirmed and could be new prognostic indicators for patients with cancer [27, 28]. The ceRNA network analysis revealed that HOXC13 was regulated by miR-31, and that KLHL40 was a target for miR-211. A research showed that “passenger" miR-31-3p was significantly upregulated in well and moderately differentiated head and neck cancers [29]. Long non-coding RNA LOC554202 promotes laryngeal squamous cell carcinoma progression through regulating miR-31.

GO and KEGG analysis showed that the DEmRNAs were mainly enriched in the functions. “Positive regulation of transcription from RNA polymerase ii promoter”, “Intracellular signal transduction” and “Sequence-specific DNA binding” and in the pathways including “HIF-1 signaling pathway” and “AMPK signaling pathway”, which are closely associated with tumorigenesis. Survival analysis showed that age, TNM stage, and clinical stage were significantly associated with survival time of OFSCC. Although unvariable Cox analysis shows that there was no correlation between smoking and survival of OFSCC, both clinical experience and scientific research recognized that smoking contributes to mortality.

This paper is the first study of ceRNA in OFSCC, a branch of HNSCC. Mature miRNA instead of RNA seq was analyzed, eliminating the interference of RNA precursor and improving the accuracy of detection. However, our study was limited that experimental verifications of the results using clinical specimens or cell lines have not been included due to insufficient material.

## Conclusion

We established a mRNA‐associated ceRNA network in OFSCC samples. The 3-mRNA model could serve as potential prognostic indicator alone or in combination with other clinicopathological for patients with OFSCC. Meanwhile, the prognostic model have been validated in the GEO database.

## Supporting information

S1 File Material of RNA and clinial. (ZIP)

S2 File The annotation of TNM stage and clinical stage. (DOCX)

S1 Table GO. (XLSX)

S2 Table KEGG. (XLSX)

S3 Table C-index. (XLSX)

S1 Fig (TIF)

S2 Fig Survival analyse of miR-31. (TIF)

S3 Fig (PDF)

S1 Text (TXT)

S2 Text (TXT)

## References

[1] Ellington TD, Henley SJ, Senkomago V, O'Neil ME, Wilson RJ, Singh S, et al Trends in Incidence of Cancers of the Oral Cavity and Pharynx—United States 2007–2016. MMWR Morb Mortal Wkly Rep. 2020;69(15):433–8. Epub 2020/04/17. 10.15585/mmwr.mm6915a1 . 32298244 PMC7755056

[2] Landis SH, Murray T, Bolden S, Wingo PA. Cancer statistics, 1999. CA Cancer J Clin. 1999;49(1):8–31, 1. Epub 1999/04/14. 10.3322/canjclin.49.1.8 . 10200775

[3] Schmidt BL, Dierks EJ, Homer L, Potter B. Tobacco smoking history and presentation of oral squamous cell carcinoma. J Oral Maxillofac Surg. 2004;62(9):1055–8. Epub 2004/09/04. 10.1016/j.joms.2004.03.010 . 15346353

[4] Salmena L, Poliseno L, Tay Y, Kats L, Pandolfi PP. A ceRNA hypothesis: the Rosetta Stone of a hidden RNA language? Cell. 2011;146(3):353–8. Epub 2011/08/02. 10.1016/j.cell.2011.07.014 21802130 PMC3235919

[5] Zhang J, Fan D, Jian Z, Chen GG, Lai PB. Cancer Specific Long Noncoding RNAs Show Differential Expression Patterns and Competing Endogenous RNA Potential in Hepatocellular Carcinoma. PLoS One. 2015;10(10):e0141042 Epub 2015/10/23. 10.1371/journal.pone.0141042 26492393 PMC4619599

[6] Aken BL, Ayling S, Barrell D, Clarke L, Curwen V, Fairley S, et al The Ensembl gene annotation system. Database (Oxford). 2016;2016 Epub 2016/06/25. 10.1093/database/baw093 27337980 PMC4919035

[7] Robinson MD, McCarthy DJ, Smyth GK. edgeR: a Bioconductor package for differential expression analysis of digital gene expression data. Bioinformatics. 2010;26(1):139–40. Epub 2009/11/17. 10.1093/bioinformatics/btp616 19910308 PMC2796818

[8] Jeggari A, Marks DS, Larsson E. miRcode: a map of putative microRNA target sites in the long non-coding transcriptome. Bioinformatics. 2012;28(15):2062–3. Epub 2012/06/22. 10.1093/bioinformatics/bts344 22718787 PMC3400968

[9] Wong N, Wang X. miRDB: an online resource for microRNA target prediction and functional annotations. Nucleic Acids Res. 2015;43(Database issue):D146–52. Epub 2014/11/08. 10.1093/nar/gku1104 25378301 PMC4383922

[10] Chou CH, Chang NW, Shrestha S, Hsu SD, Lin YL, Lee WH, et al miRTarBase 2016: updates to the experimentally validated miRNA-target interactions database. Nucleic Acids Res. 2016;44(D1):D239–47. Epub 2015/11/22. 10.1093/nar/gkv1258 26590260 PMC4702890

[11] Huang da W, Sherman BT, Lempicki RA. Systematic and integrative analysis of large gene lists using DAVID bioinformatics resources. Nat Protoc. 2009;4(1):44–57. Epub 2009/01/10. 10.1038/nprot.2008.211 . 19131956

[12] Alhamzawi R, Ali HTM. The Bayesian adaptive lasso regression. Math Biosci. 2018;303:75–82. Epub 2018/06/20. 10.1016/j.mbs.2018.06.004 . 29920251

[13] Ren S, Huang S, Ye J, Qian X. Safe Feature Screening for Generalized LASSO. IEEE Trans Pattern Anal Mach Intell. 2018;40(12):2992–3006. Epub 2018/07/11. 10.1109/TPAMI.2017.2776267 . 29990186

[14] Waldmann P, Ferencakovic M, Meszaros G, Khayatzadeh N, Curik I, Solkner J. AUTALASSO: an automatic adaptive LASSO for genome-wide prediction. BMC Bioinformatics. 2019;20(1):167 Epub 2019/04/04. 10.1186/s12859-019-2743-3 30940067 PMC6444607

[15] Zhou L, Tang L, Song AT, Cibrik DM, Song PX. A LASSO Method to Identify Protein Signature Predicting Post-transplant Renal Graft Survival. Stat Biosci. 2017;9(2):431–52. Epub 2018/02/06. 10.1007/s12561-016-9170-z 29399205 PMC5793946

[16] Barabasi AL, Oltvai ZN. Network biology: understanding the cell's functional organization. Nat Rev Genet. 2004;5(2):101–13. Epub 2004/01/22. 10.1038/nrg1272 . 14735121

[17] Wang Y, Hou J, He D, Sun M, Zhang P, Yu Y, et al The Emerging Function and Mechanism of ceRNAs in Cancer. Trends Genet. 2016;32(4):211–24. Epub 2016/02/29. 10.1016/j.tig.2016.02.001 26922301 PMC4805481

[18] Qi X, Zhang DH, Wu N, Xiao JH, Wang X, Ma W. ceRNA in cancer: possible functions and clinical implications. J Med Genet. 2015;52(10):710–8. Epub 2015/09/12. 10.1136/jmedgenet-2015-103334 . 26358722

[19] Turley RS, Finger EC, Hempel N, How T, Fields TA, Blobe GC. The type III transforming growth factor-beta receptor as a novel tumor suppressor gene in prostate cancer. Cancer Res. 2007;67(3):1090–8. Epub 2007/02/07. 10.1158/0008-5472.CAN-06-3117 . 17283142

[20] Copland JA, Luxon BA, Ajani L, Maity T, Campagnaro E, Guo H, et al Genomic profiling identifies alterations in TGFbeta signaling through loss of TGFbeta receptor expression in human renal cell carcinogenesis and progression. Oncogene. 2003;22(39):8053–62. Epub 2003/09/13. 10.1038/sj.onc.1206835 . 12970754

[21] Bandyopadhyay A, Lopez-Casillas F, Malik SN, Montiel JL, Mendoza V, Yang J, et al Antitumor activity of a recombinant soluble betaglycan in human breast cancer xenograft. Cancer Res. 2002;62(16):4690–5. Epub 2002/08/17. . 12183427

[22] Bandyopadhyay A, Zhu Y, Cibull ML, Bao L, Chen C, Sun L. A soluble transforming growth factor beta type III receptor suppresses tumorigenicity and metastasis of human breast cancer MDA-MB-231 cells. Cancer Res. 1999;59(19):5041–6. Epub 1999/10/16. . 10519421

[23] Bragado P, Estrada Y, Parikh F, Krause S, Capobianco C, Farina HG, et al TGF-beta2 dictates disseminated tumour cell fate in target organs through TGF-beta-RIII and p38alpha/beta signalling. Nat Cell Biol. 2013;15(11):1351–61. Epub 2013/10/29. 10.1038/ncb2861 24161934 PMC4006312

[24] Bandyopadhyay A, Wang L, Lopez-Casillas F, Mendoza V, Yeh IT, Sun L. Systemic administration of a soluble betaglycan suppresses tumor growth, angiogenesis, and matrix metalloproteinase-9 expression in a human xenograft model of prostate cancer. Prostate. 2005;63(1):81–90. Epub 2004/10/07. 10.1002/pros.20166 . 15468171

[25] Wei Z, Chang K, Fan C. Hsa_circ_0042666 inhibits proliferation and invasion via regulating miR-223/TGFBR3 axis in laryngeal squamous cell carcinoma. Biomed Pharmacother. 2019;119:109365 Epub 2019/09/17. 10.1016/j.biopha.2019.109365 . 31525642

[26] Bhosale PG, Cristea S, Ambatipudi S, Desai RS, Kumar R, Patil A, et al Chromosomal Alterations and Gene Expression Changes Associated with the Progression of Leukoplakia to Advanced Gingivobuccal Cancer. Transl Oncol. 2017;10(3):396–409. Epub 2017/04/24. 10.1016/j.tranon.2017.03.008 28433800 PMC5403767

[27] Garg A, O'Rourke J, Long C, Doering J, Ravenscroft G, Bezprozvannaya S, et al KLHL40 deficiency destabilizes thin filament proteins and promotes nemaline myopathy. J Clin Invest. 2014;124(8):3529–39. Epub 2014/06/25. 10.1172/JCI74994 24960163 PMC4109545

[28] Rundqvist HC, Montelius A, Osterlund T, Norman B, Esbjornsson M, Jansson E. Acute sprint exercise transcriptome in human skeletal muscle. PLoS One. 2019;14(10):e0223024 Epub 2019/10/28. 10.1371/journal.pone.0223024 31647849 PMC6812755

[29] Yang S, Wang J, Ge W, Jiang Y. Long non-coding RNA LOC554202 promotes laryngeal squamous cell carcinoma progression through regulating miR-31. J Cell Biochem. 2018;119(8):6953–60. Epub 2018/05/09. 10.1002/jcb.26902 . 29737563

