# Supplementary figures and images for: Construction and analysis of a competing endogenous RNA network to reveal potential prognostic biomarkers for Oral Floor Squamous Cell Carcinoma
Source: PLoS One. 2020 Sep 15;15(9):e0238420. doi: 10.1371/journal.pone.0238420 (PMC7491744; doi:10.1371/journal.pone.0238420)

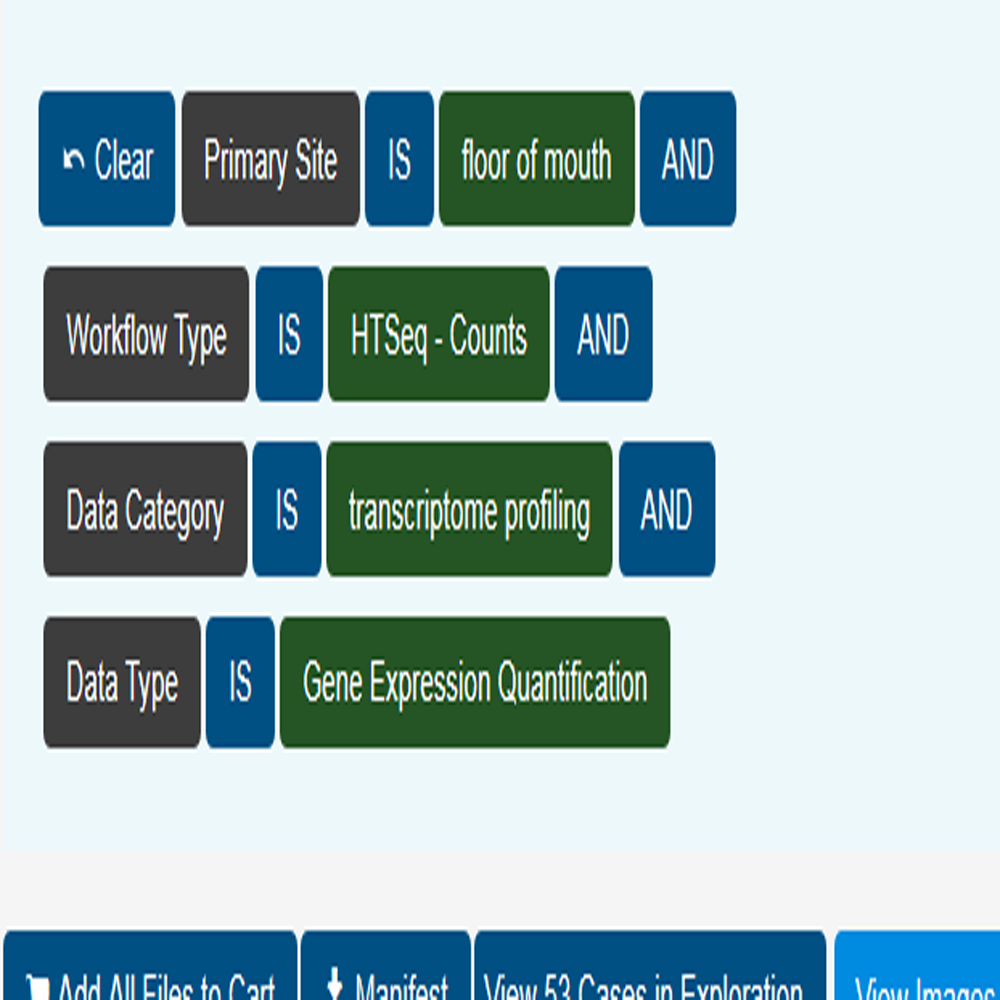

Supplement: S1 File — (ZIP) [file pone.0238420.s001.zip › 4.search strategy2(mRNA lncRNA).tif]

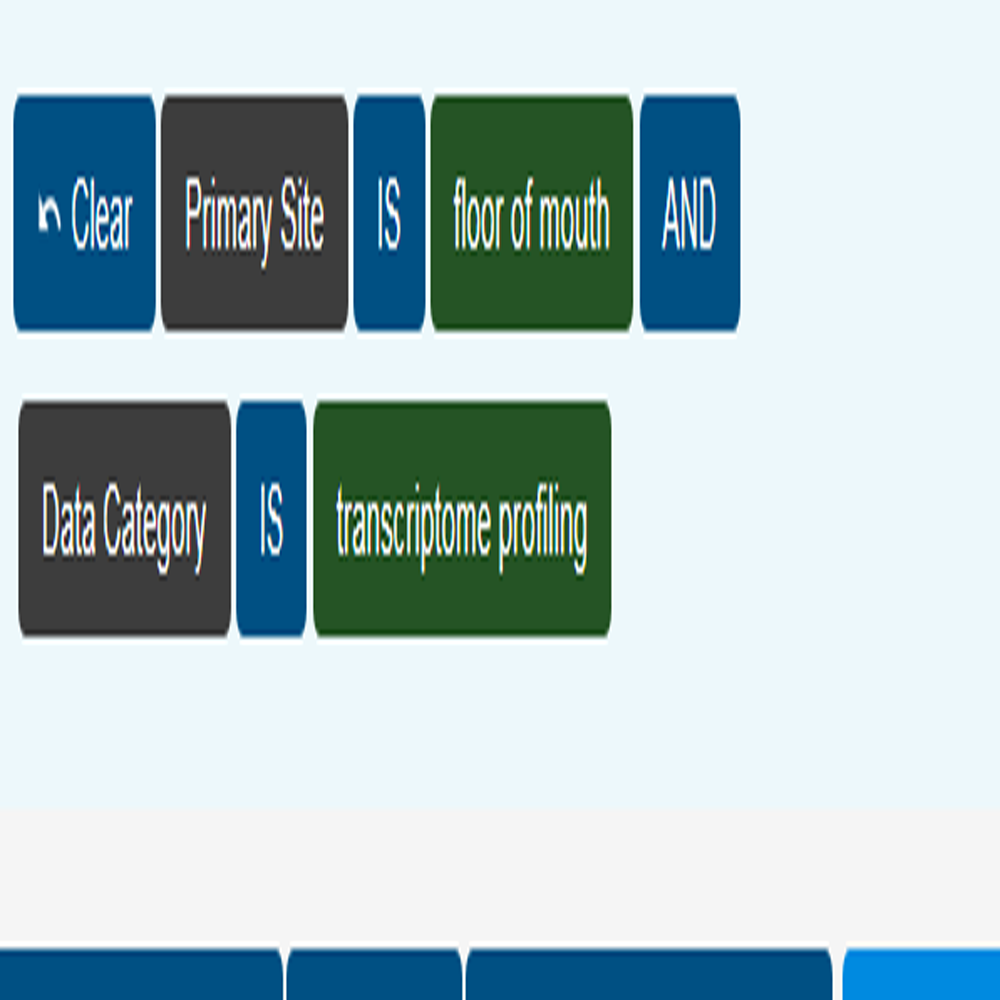

Supplement: S1 File — (ZIP) [file pone.0238420.s001.zip › 5.search strategy4(mature miRNA).tif]

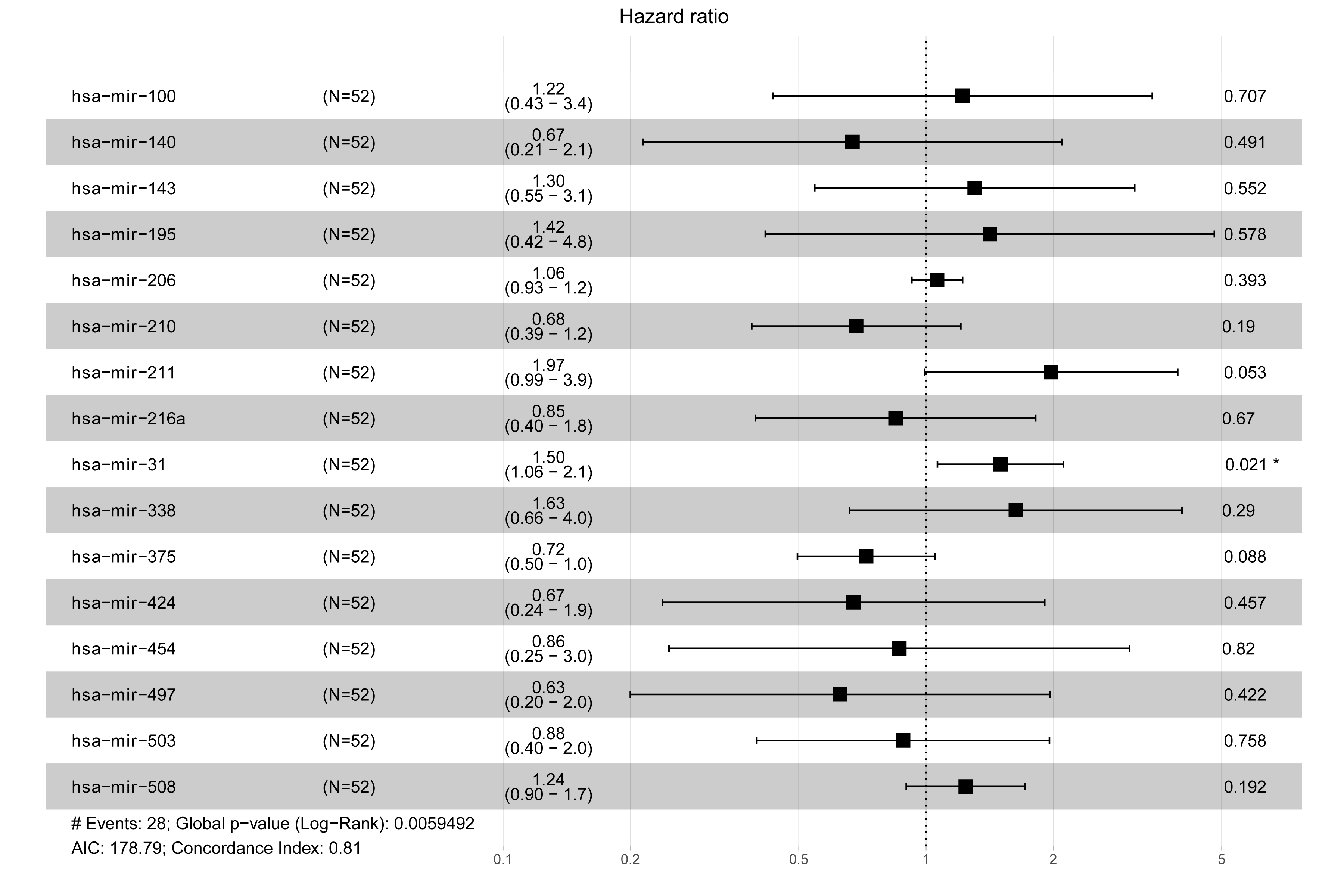

Supplement: S1 Fig — (TIF) [file pone.0238420.s006.tif]

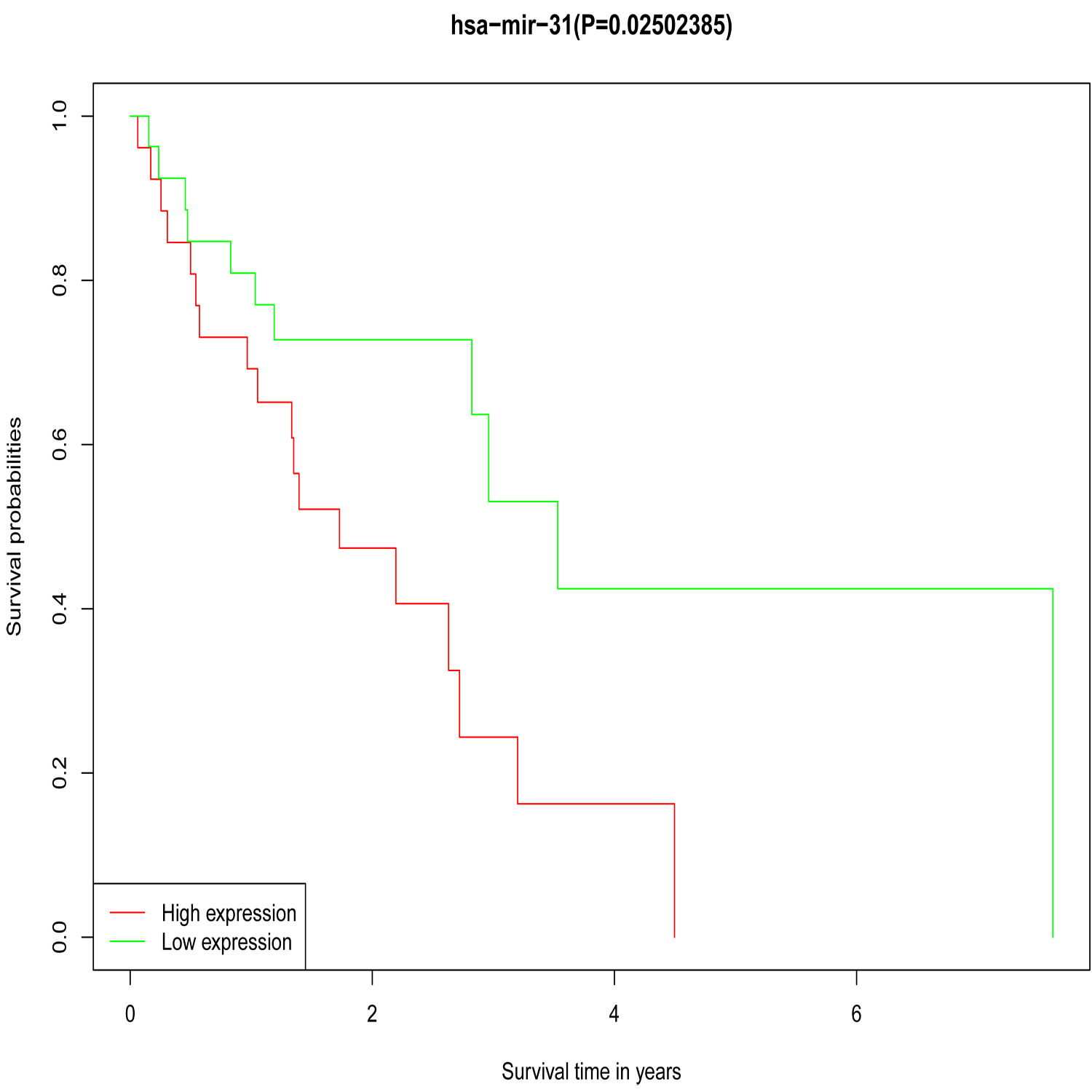

Supplement: S2 Fig — (TIF) [file pone.0238420.s007.tif]

# Hazard ratio

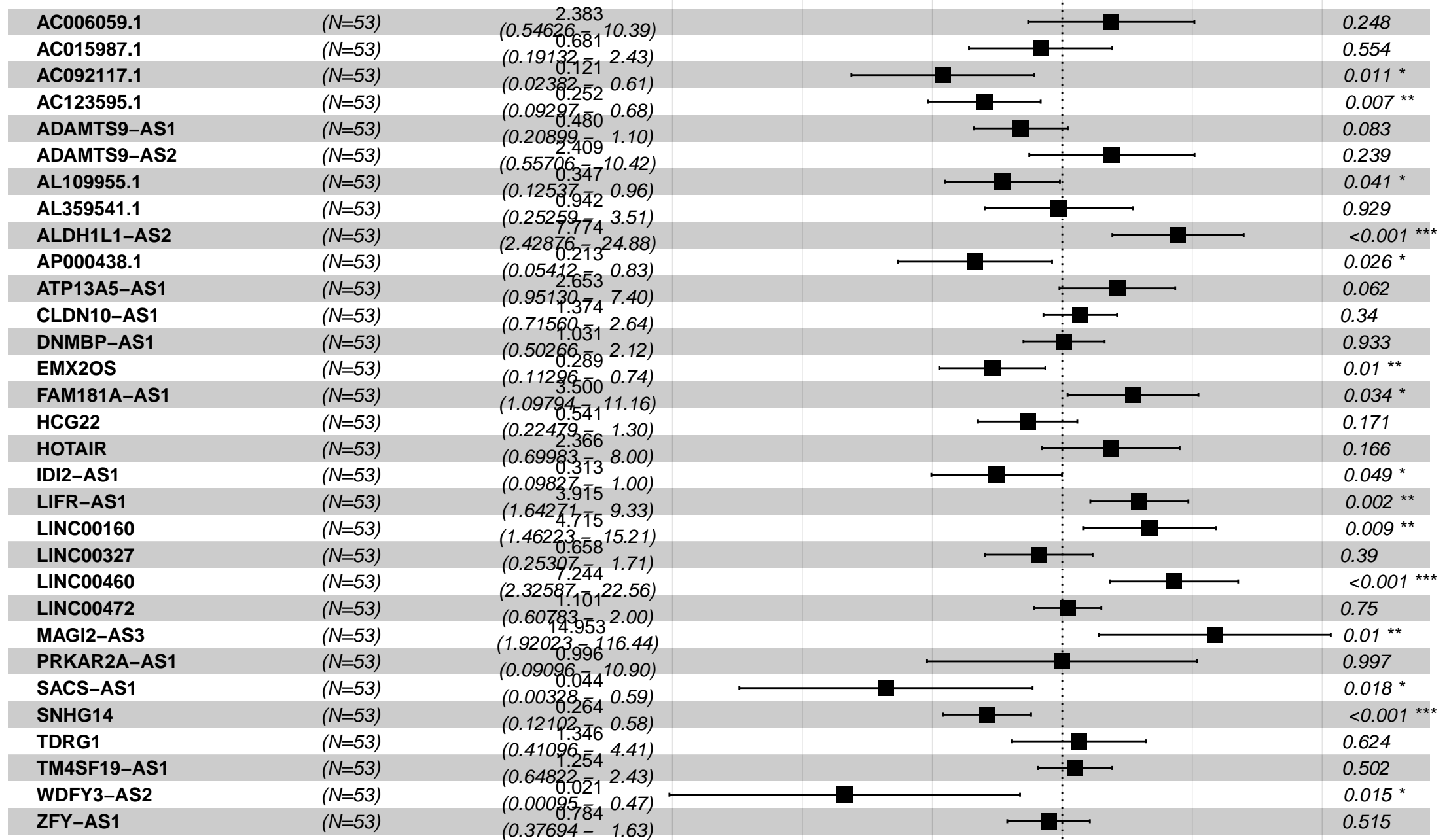

# Events: 29; Global p-value (Log-Rank): 0.015482

AIC: 195.22; Concordance Index: 0.89

Supplement: S3 Fig — (PDF) [file pone.0238420.s008.pdf]
