# Supplementary material for: Construction and analysis of a competing endogenous RNA network to reveal potential prognostic biomarkers for Oral Floor Squamous Cell Carcinoma
Source: PLoS One. 2020 Sep 15;15(9):e0238420. doi: 10.1371/journal.pone.0238420 (PMC7491744; doi:10.1371/journal.pone.0238420)
Supplement: S2 File — (DOCX) [file pone.0238420.s002.docx]

The annotation of TNM stage and clinical stage [32]: TNM staging of oral cancer is an important index for clinical diagnosis, treatment and prognosis evaluation of cancer patients, and also plays a major role in doctor-patient communication. TNM staging system is the most common tumor staging system in the world. It was proposed by Pierre Denoix in 1943-1952. After that, the American Joint Committee on cancer (AJCC) and the Union for international cancer control (UICC) gradually improved it, and established an international staging standard.

TNM staging system mainly includes three grading indexes：

“T” refers to the size of the primary tumor and the involvement of surrounding tissues, T1-4 refers to the size and / or range of the primary tumor, This refers to the early tumor without dissemination, TX refers to the primary tumor which can not test;

“N” refers to the regional lymph node involvement containing N1 N2 N3. The regional lymph node classification system of various tumors is different;

“M” refers to distant metastasis of tumor. Mo refers to no metastasis, M1 refers to tumor spread to other parts of the body.

Clinically, the diagnosis of oral cancer patients is based primarily on pathological and imaging diagnosis. The T stage of the tumor obtained during the auxiliary clinical examination or operation, and then the condition of tumor patients was comprehensively evaluated and the next treatment was guided.Studies have demonstrated that 50%-70% of patients with oral cancer have lymph node metastasis at the initial diagnosis (N>0), and their 5-year survival rate is almost half lower than that of patients without lymph node metastasis.

TNM staging is different in disease systems, but it depends on T, N and M scores to get the corresponding comprehensive clinical staging, i.e. stage I (early stage), stage II-III (progressive stage), stage IV (late stage), etc.

According to the size of the primary focus, the status of lymph node metastasis and whether there is distant metastasis, the oral floor cancer can comprise four stages. We accept it is generally that stage Ⅰand stage Ⅱ tumors are localized and have a relatively shallow infiltration range. The tumor size should be lower than 4cm, without invasion of adjacent tissues, without lymph node metastasis. These tumors belong to stage I and stage II, which belongs to the early stage of oral floor cancer. Patients with stage III and stage IV often have lymph node metastasis, or invasion of adjacent tongue tissue, jaw or distant metastasis. The earlier the stage, the better the prognosis.

Reference：

1. Ricardi U, et al. Eighth Edition of the UICC Classification of Malignant Tumours: an overview of the changes in the pathological TNM classification criteria-What has changed and why? VIRCHOWS ARCH. 2018-04-01;472(4):519-31.
